# Two Dimensional Yau-Hausdorff Distance with Applications on Comparison of DNA and Protein Sequences

**DOI:** 10.1371/journal.pone.0136577

**Published:** 2015-09-18

**Authors:** Kun Tian, Xiaoqian Yang, Qin Kong, Changchuan Yin, Rong L. He, Stephen S.-T. Yau

**Affiliations:** 1 Department of Mathematical Sciences, Tsinghua University, Beijing 100084, China; 2 Department of Mathematics, Statistics and Computer Science, The University of Illinois at Chicago, Chicago, IL 60607-7045, United States of America; 3 Department of Biological Sciences, Chicago State University, Chicago, IL 60628, United States of America; University of Michigan, UNITED STATES

## Abstract

Comparing DNA or protein sequences plays an important role in the functional analysis of genomes. Despite many methods available for sequences comparison, few methods retain the information content of sequences. We propose a new approach, the Yau-Hausdorff method, which considers all translations and rotations when seeking the best match of graphical curves of DNA or protein sequences. The complexity of this method is lower than that of any other two dimensional minimum Hausdorff algorithm. The Yau-Hausdorff method can be used for measuring the similarity of DNA sequences based on two important tools: the Yau-Hausdorff distance and graphical representation of DNA sequences. The graphical representations of DNA sequences conserve all sequence information and the Yau-Hausdorff distance is mathematically proved as a true metric. Therefore, the proposed distance can preciously measure the similarity of DNA sequences. The phylogenetic analyses of DNA sequences by the Yau-Hausdorff distance show the accuracy and stability of our approach in similarity comparison of DNA or protein sequences. This study demonstrates that Yau-Hausdorff distance is a natural metric for DNA and protein sequences with high level of stability. The approach can be also applied to similarity analysis of protein sequences by graphic representations, as well as general two dimensional shape matching.

## Introduction

Comparison of DNA sequences or protein sequences is a problem that has been studied in biological sciences for years. It is an important mean to understand the nature of known proteins and predict the unknown functions of the sequences. Many approaches have been proposed for measuring the similarity between DNA sequences and protein sequences, including multiple sequence alignment [1], moment vectors [2] and feature vectors [3]. Multiple sequence alignment uses dynamic programming techniques to identify the globally optimal alignment solution, and is the most popular method in sequence comparison. However, the sequence alignment problem is NP-hard, making it infeasible for studying large data-sets. The moment vector approach characterizes the DNA space by assigning each DNA sequence a vector consisting of moments obtained from its graphical curve. The distance between sequences is then defined to be the Euclidean distance between their corresponding vectors. This approach is effective and operates in linear time. There is no criterion yet to determine the dimension of the moment vector, and the method does not present the DNA or protein space accurately, as we will show in this paper. On the other hand, it is obvious that the correspondence between feature vectors and DNA sequences is not one-to-one. Thus, the feature vector method is not reliable due to loss of information about nucleotide. New methods on sequence comparisons are being continuously developed. For example, Liu et al developed the Python package for generating various modes of feature vectors for sequences [4]. This method depends on fifteen types of feature vectors of sequence, which can be extremely large for computing DNA sequences of long lengths. Zou et al proposed the centre star MSA strategy for sequence alignment [5]. It offers new tools to address large-scale data for multiple sequence alignment.

In this article, we establish a new approach to measure the distance between DNA (or protein) sequences: the Yau-Hausdorff method. This study arises from the graphical representation of DNA or protein sequences proposed by Yau [2, 6], in which each DNA or protein sequence is represented by a curve in two-dimensional plane. The graphical representation method results in one-to-one mapping between DNA sequences and the graphical curves. However the question on how to measure the true distance between two DNA curves has not been addressed up to now. The main contribution of this study is to introduce a new distance between two dimensional curves defined by the DNA (or protein) sequences.

Although many techniques for two dimensional distance are available, presently the most useful criterion to measure the similarity between two-dimensional point sets is the Hausdorff distance [7, 8]. This distance can be used to determine the degree of resemblance between two point sets that are superimposed. However the general Hausdorff distance does not satisfy our requirements, since we wish to measure the minimum distance between two point sets under rigid motions including translation and rotation. The minimum Hausdorff distance under rigid motions is a well-defined metric and not only measure the distance of two point sets, but also the similarity of their shapes. Mathematicians have tried to find efficient algorithms to compute this distance, but none of the existing algorithms reaches the level of efficiency required for analyzing long DNA (or protein) sequences.

In this article, we define the Yau-Hausdorff distance, a new metric which measures the similarity between two-dimensional point sets. This new metric possesses some advantages: it is a well-defined metric in mathematics; it is a natural generalization of the minimum one-dimensional Hausdorff distance; it takes translation and rotation into full consideration; and it is much more efficient to compute than the existing two-dimensional minimum Hausdorff distance. These advantages enable it to be a powerful tool for comparing two-dimensional point sets, particularly for comparing DNA or protein sequences.

In the first section, we introduce two important methods: the Yau-Hausdorff distance and the graphical representation of DNA (or protein) sequences. In the second section, results from applying the Yau-Hausdorff method to several biological examples are presented and compared with results achieved by previous approaches. In the third section we discuss the advantages of the Yau-Hausdorff method and its broader applications.

## Methods

### Graphical representation of DNA sequences

**Fig 1:**
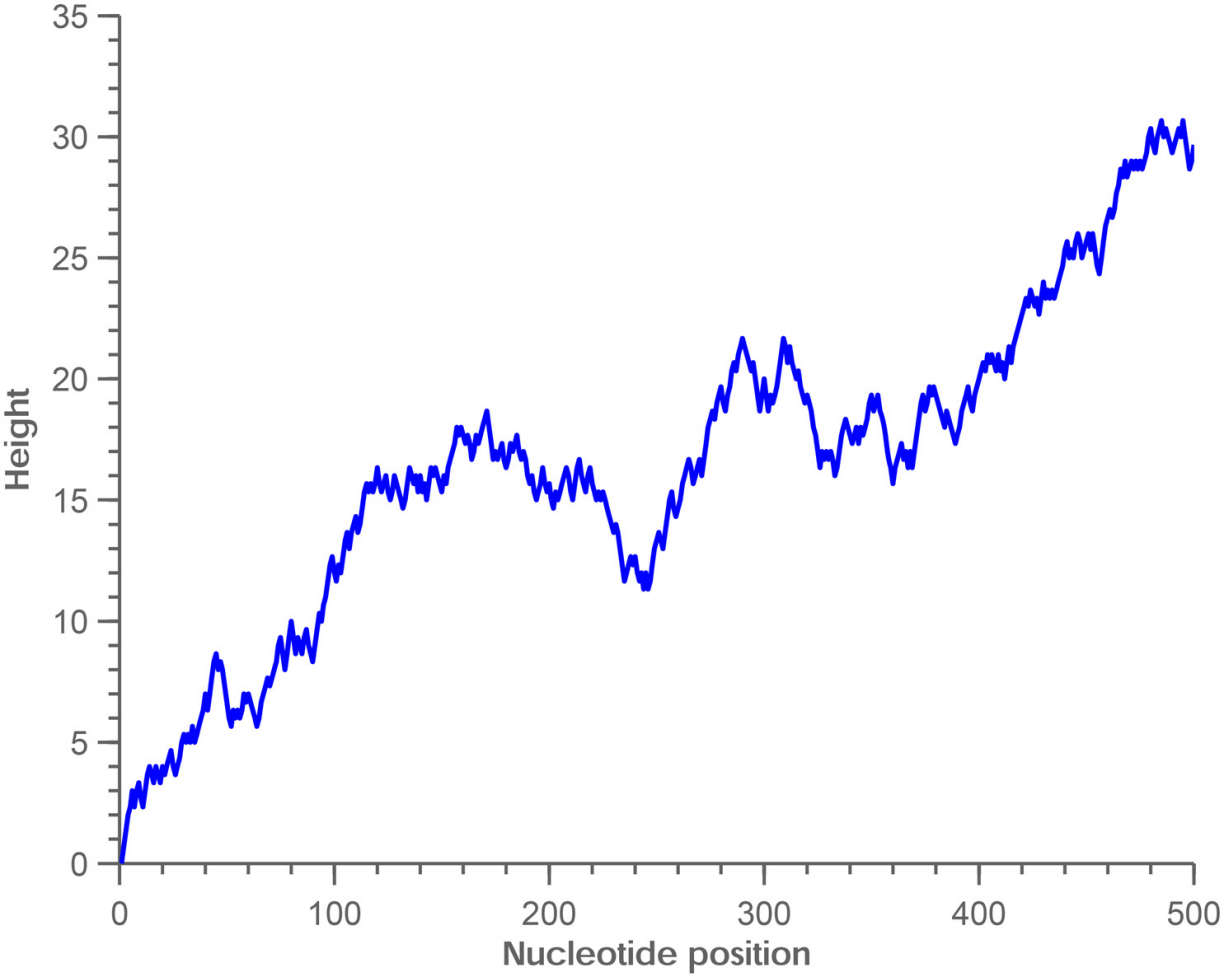
Graphical representation of human mitochondrial DNA (1–500 bp, GenBank:X93334).

Yau proposed a unique method to represent DNA sequences by a two dimensional graph [6]. Compared with previous methods, the graphical representation resolves sequence degeneracy and is proven to eliminate circuit formation. We use Yau’s method to generate graphical representations of DNA sequences. We chose following four vectors to represent the four nucleotides A, G, C and T respectively: (1,2/3) → *T*, (1,1/3) → *A*, (1, −1/3) → *C*, (1, −2/3) → *G*. An illustrative example is given in Fig 1 for the graphic representation of the first 500 bp human mtochondrial DNA sequence.

### Graphical representation of protein sequences

We use the approach proposed by Yau in 2008 [2] to generate graphical representations of protein sequences. Here is a brief description of the process.

A protein sequence is a string composed of 20 fundamental amino acids. Fauchere and Pliska assigned a value to each of these 20 amino acids according to its hydrophobicity [9], and Yau [2] mapped each value to a number between -1 and 1, such that the 20 numbers are uniformly distributed on the positive and the negative axes respectively.

Having determined the correspondence between each amino acid i and a real number *y*_*i*_ ∈ [−1,1], a vector can be defined with the horizontal component 1 and the vertical component *y*_*i*_. Given a protein sequence, its graphical representation is the collection of the corresponding vectors of the amino acids in the protein sequence, i.e., a point set with the size *l*+1, where *l* is the sequence length.

The construction procedure for the graphical representation is natural in that it only collects information based on the hydrophobicity scale of the 20 amino acids and the protein sequence, and the information is then transformed into an intuitive two-dimensional graph, which naturally reflects the biological characteristics of a protein.

Now that we have the graphical representation of DNA(or protein) sequences, all we need is a criterion that measures the similarity between two curves to characterize similarity of sequences. Huang proposed an approach using the feature vector as the numerical characterization of a DNA sequence [3]. The feature vector consists of a 10-dimensional vector formed from the tallest peak, the lowest point, and the central points of the graphical representation of a DNA sequence. However, the feature vector may not preserve the complete information in a graphical representation because the feature vector does not contain enough information to reconstruct the curve, and thus cannot fully represent the distribution of nucleotides in a DNA sequence. Furthermore, translations or rotations of the curves are not considered in the method, so the feature vector approach may be unreliable in DNA sequence comparison.

Our approach differs from the feature vector method in that we compare DNA or protein sequences by measuring the similarity between graphical representations directly, so our approach does not lose information within DNA or protein sequences. In addition, we take translation and rotation into account when making comparisons. To accomplish this, we propose a new criterion for two-dimensional point set comparison, the Yau-Hausdorff distance.

### Minimum two-dimensional Hausdorff distance

We first introduce Hausdorff distance, one of the most widely used criteria for point set comparisons [7]. For two point sets A and B, the Hausdorff distance between point A and point B sets is defined by

$$h\left(A,B\right)=\max\{\max_{a\in A}\min_{b\in B}|a-b|,\max_{b\in B}\min_{a\in A}|b-a|\}$$ (1)

Intuitively, this can be considered as the minimum distance for which at least one point in set B is accessible from any point of set A, and vice versa.

When comparing graphical representations of DNA or protein sequences, we emphasize the level of shape similarity. Thus, the ideal metric should consider the optimal fit under rigid motions including translation and rotation. Since the general Hausdorff distance measures the distance between two fixed sets, it shall not be a good candidate for an ideal metric although the general Hausdorff distance is a defined metric.

The minimum two-dimensional Hausdorff distance as defined below is a well-defined metric that indeed meets this requirement.

$$H^{2}\left(A,B\right)=\min_{\theta \in [0,2\pi ]}\min_{t\in R^{2}}h\left(A+t,B^{\theta }\right)$$ (2)

where *h* is Hausdorff distance defined in Eq (1) and *h*(*A*+*t*,*B* ^*θ*^) stands for the Hausdorff distance between A and B after shifting A rightward by t and rotating B counterclockwise by *θ*.

The minimum two-dimensional Hausdorff distance is widely used in graph comparison, and several algorithms have been proposed for this central distance. Some of the algorithms rely on the assumption that there are only grid points in the point sets. These algorithms are mainly used in pixel image matching such as photo identification and MRI analysis. But as comparing graphical representations of sequences requires precise rotation of each point, clearly the grid point assumption is not satisfied. The best matching of two shapes under translation and rotation can be obtained by the minimum Hausdorff distance. For two point sets with size m and n, the time complexity of the minimum Hausdorff distance by the Huttenlocher algorithm is *O*((*m*+*n*)^6^log(*mn*)) [10]. That algorithm for minimum Hausdorff distance under Euclidean motion was improved later with the complexity as *O*((*m*+*n*)^5^log^2^ *mn*)) [8]. However, these algorithms are still not feasible for comparing graphic curves of long DNA (or protein) sequences of more than 10000 bp. These limitations highlight the need to improve the algorithm of minimum Hausdorff distance. In this study, we present a new metric, the Yau-Hausdorff distance, for matching two-dimensional curves under translation and rotation.

### Yau-Hausdorff distance

We propose here the Yau-Hausdorff distance in terms of the minimum one-dimensional Hausdorff distance [11]. The minimum Hausdorff distance between two one-dimensional point sets A and B under translation is defined as

$$H^{1}\left(A,B\right)=\min_{t\in R}h\left(A+t,B\right)$$ (3)

where *h*(*A*+*t*,*B*) is the Hausdorff distance between A and B after shifting A rightward by t. This equation can be rewritten as

$$H^{1}\left(A,B\right)=\min_{t\in R}\max\{\max_{a\in A+t}\min_{b\in B}|a-b|,\max_{b\in B}\min_{a\in A+t}\left|b-a|\right\}$$ (4)

The Yau-Hausdorff distance is then defined in terms of *H* ^1^(*A*,*B*):

$$\begin{array}{c} D\left(A,B\right)=\max\{\max_{\theta }\min_{\varphi }H^{1}\left(P_{x}\left(A^{\theta }\right),P_{x}\left(B^{\varphi }\right)\right),\\ \max_{\varphi }\min_{\theta }H^{1}\left(P_{x}\left(A^{\theta }\right),P_{x}\left(B^{\varphi }\right)\right)\} \end{array}$$ (5)

where *P*_*x*_(*A* ^*θ*^) is an one-dimensional point set representing the projection of A on the x-axis after being rotated counterclockwise by *θ*.

The Yau-Hausdorff distance D defined above possesses the following properties:

1. D can be proven as an metric (the proof is available in the supplementary materials).
2. D is defined in terms of and inherits properties from the minimum one-dimensional Hausdorff distance. It is so far the most accurate criterion for two-dimensional point set comparison.
3. D fully considers all translation and rotation in the two-dimensional space.
4. Using the projection of two-dimensional point sets, D successfully avoids calculation of the Hausdorff distance of two-dimensional sets, and can be computed efficiently.

The Yau-Hausdorf distance is not equal to the two-dimensional minimum Hausdorff distances. In fact, the Yau-Hausdorf distance is the lower bound of the minimum two-dimensional Hausdorff distances. The proof that *H* ^2^(*A*,*B*) ≥ *D*(*A*,*B*) and an example showing this inequality are provided in the supplementary materials.

Our algorithm to compute the Yau-Hausdorff distance D is as follows:

Let *A* = {*a*_1_, *a*_2_, …, *a*_*n*_} ⊂ ℝ^3^, *B* = {*b*_1_, *b*_2_, …, *b*_*m*_} ⊂ ℝ^3^.

1. Fix *a*_1_. For *i* = 2, 3, …, *n* rotate A such that *a*_1_ *a*_*i*_//*x*−*axis*. We get *θ*_1_, *θ*_2_, …, *θ*_*n*−1_.
2. Fix *a*_2_. For *i* = 3, 4, …, *n* rotate A such that *a*_2_ *a*_*i*_//*x*−*axis*. We get *θ*_*n*_, *θ*_*n*+1_, …, *θ*_2*n*−3_.
3. Fix *a*_3_. For *i* = 4, 5, …, *n* rotate A such that *a*_3_ *a*_*i*_//*x*−*axis*. We get *θ*_2*n*−2_, *θ*_2*n*−1_, …, *θ*_3*n*−6_
4. Randomly rotate 1000 times by $\theta_{1}^{\prime },\theta_{2}^{\prime },...,\theta_{1000}^{\prime }$.
5. Let the set of these rotations be $M=\{\theta_{1},\theta_{2},...,\theta_{3n-6},\theta_{1}^{\prime },\theta_{2}^{\prime },...,\theta_{1000}^{\prime }\}$.
6. Similarly we get the set of rotations for B $N=\{\varphi_{1},\varphi_{2},...,\varphi_{3m-6},\varphi_{1}^{\prime },\varphi_{2}^{\prime },...,\varphi_{1000}^{\prime }\}$
7. For each *θ* ∈ *M*, we compute min_*φ* ∈ *N*_ *H* ^1^(*P*_*x*_(*A* ^*θ*^),*P*_*x*_(*B* ^*φ*^)).
8. For all *θ* ∈ *M*, we get *D*_1_ = max_*θ* ∈ *M*_min_*φ* ∈ *N*_ *H* ^1^(*P*_*x*_(*A* ^*θ*^),*P*_*x*_(*B* ^*φ*^)).
9. Similarly we get *D*_2_ = max_*φ* ∈ *N*_ min_*θ* ∈ *M*_ *H* ^1^(*P*_*x*_(*A* ^*θ*^),*P*_*x*_(*B* ^*φ*^)).
10. Take *D*(*A*,*B*) = max{*D*_1_,*D*_2_}.

It shall be noted that the algorithms for calculating the one-dimensional minimum Hausdorff distance include the algorithm proposed by G.Rote in 1991 [11] and the improved algorithm proposed by Li [12]. Because of the improved efficiency in Li’s algorithm, we chose the Li’s algorithm for computing the Yau-Hausdorff distance.

### Similarity analysis of DNA or protein sequences by the Yau-Hausdorff distance

Using the Yau-Hausdorff distance of graphic curves, similarity analysis of DNA or protein sequences is performed by clustering the sequences into phylogenetic trees. The dissimilarity matrix of given sequences is constructed from the Yau-Hausdorff distance of pairwise sequences. UPGMA (Unweighted Pair Group Method with Arithmetic Mean) hierarchical clustering method from a pairwise distance matrix is used to construct phylogenetic trees [13]. The resulting UPGMA tree reflects the structure and relationship of the sequences presented in the distance matrix.

## Results

### DNA sequence comparison

We apply the Yau-Hausdorff method by comparing the DNA sequences of the COI genes, barcoding, H1N1, and the Influenza virus neuraminidase (NA) genes to verify the accuracy of our method on its ability to cluster genomes. GenBank access numbers of DNA and protein sequences used in this study are listed in S2 File.

#### COI dataset analysis

**Fig 2:**
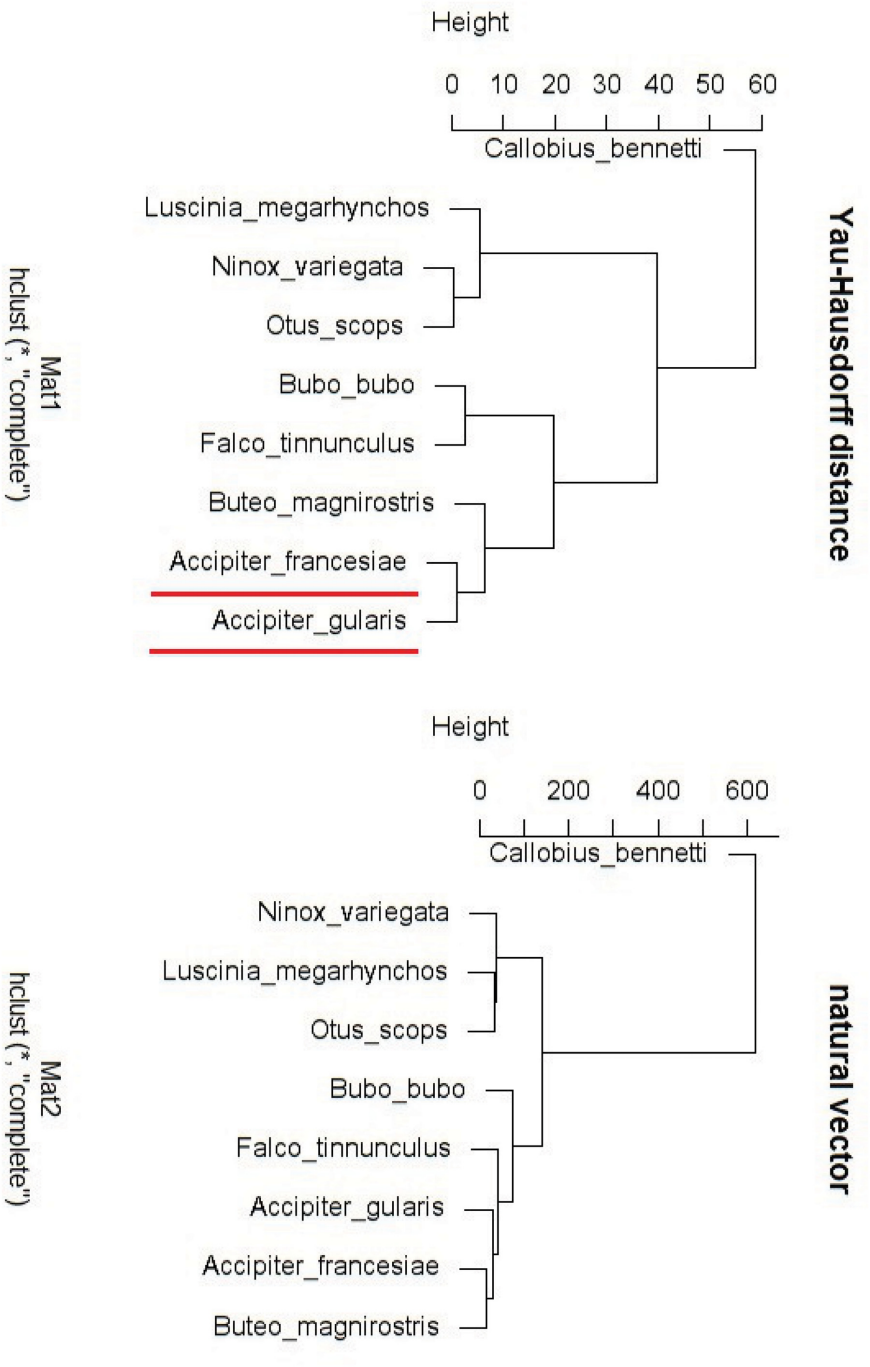
Hierarchical tree of COI sequences (Yau-Hausdorff method and natural vector method).

Paul D. N. Hebert [14] claimed that the mitochondrial gene cytochrome c oxidase I (COI) can serve as the core of a global bioidentification system for animals [14]. First we applied our method on the COI gene of nine species, including one spider and eight raptor. The average length of COI gene sequences is about 700 bp. These nine sequences are transferred to graphical representations using Yau’s method [6], then the Yau-Hausdorff distance between each two graphs is computed. We get the distance matrix and generated the corresponding hierarchical tree. We also use the natural vector method [15] to test the result. The distance provided by natural vector method is Euclidean distance of the vectors presented by DNA sequences in 12-dimensional space *R* ^12^, while Yau-Hausdorff method is based on calculating the minimum Hausdorff distance of point sets coming from the graphical representation of sequences. The clustering results of both the Yau-Hausdorff method and the natural vector method are compared as shown in Fig 2.

Callobius bennetti is a spider which should be separated from those eight raptor. Fig 2 demonstrates that both methods successfully cluster Callobius bennetti outside the cluster of raptor. In addition, the Yau-Hausdorff method clusters Accipiter francesiae and Accipiter gularis closer than the natural vector method. According to biological classification, Accipiter francesiae and Accipiter gularis belong to the same genus Accipiter. Therefore, it is reasonable that these two species are closer to each other than other species. In this case, Yau-Hausdorff method is more reliable than the natural vector method.

#### Barcoding DNA analysis

**Fig 3:**
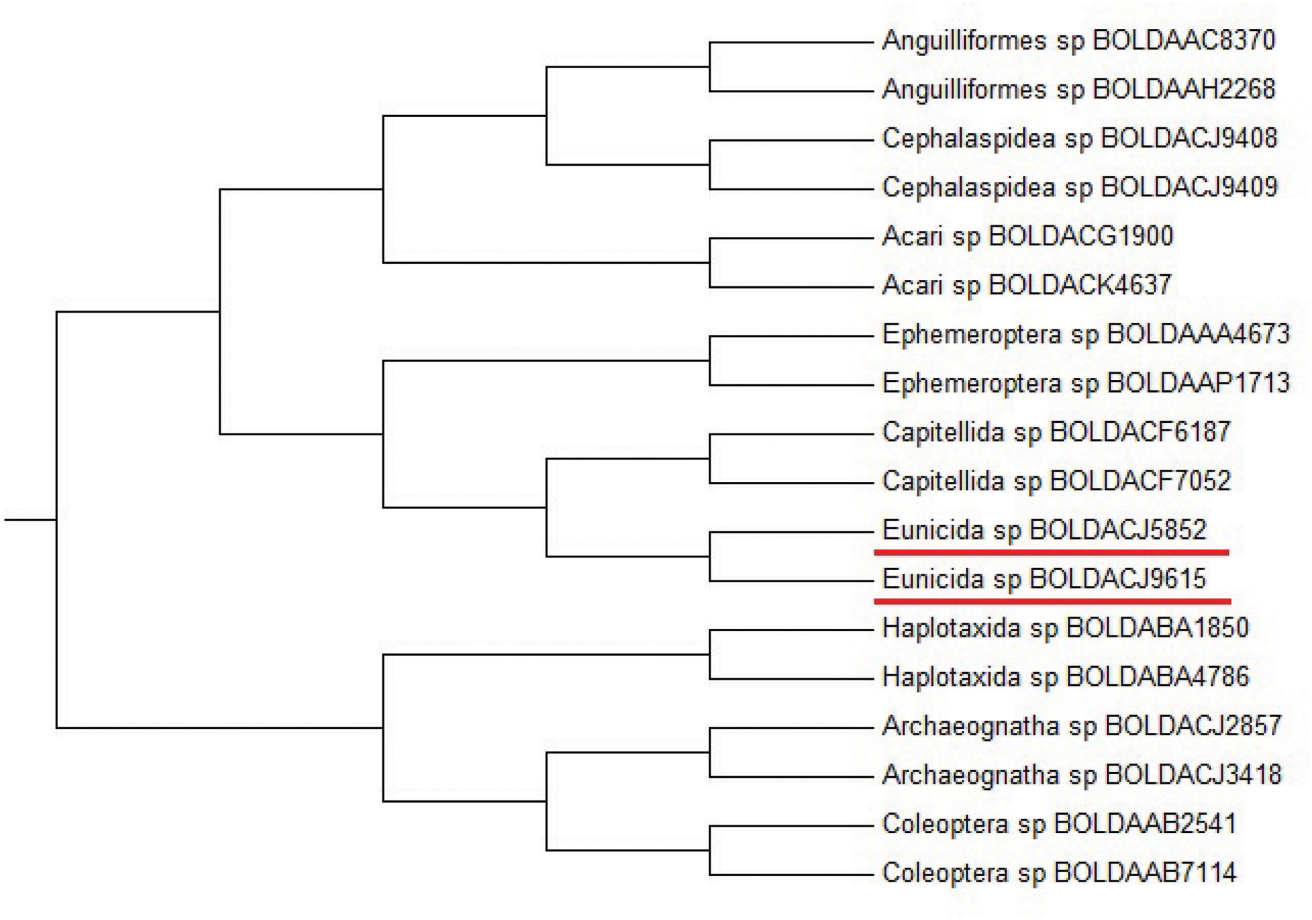
Hierarchical tree of barcoding DNA sequences (Yau-Hausdorff method).

**Fig 4:**
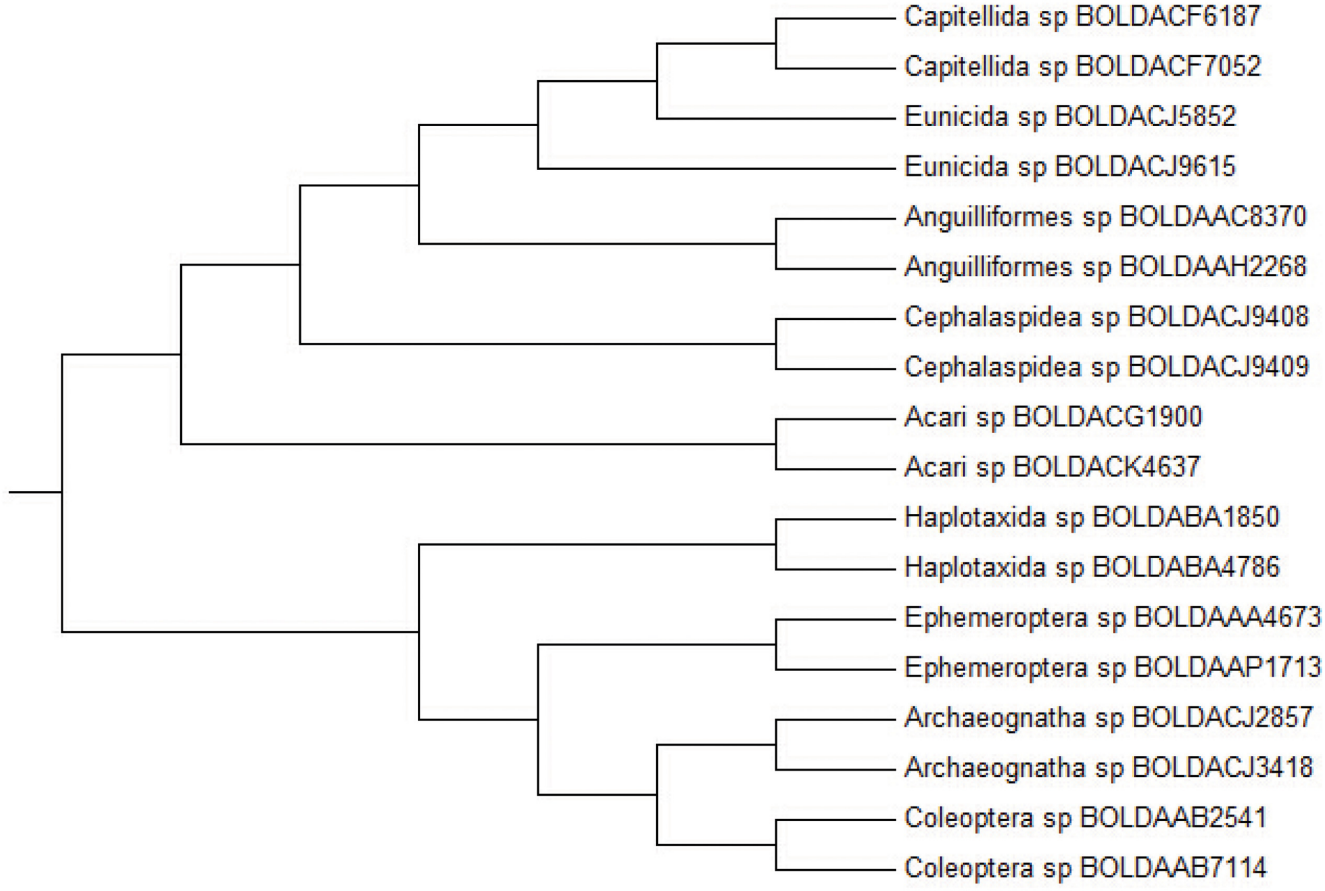
Hierarchical tree of barcoding DNA sequences (Feature vector method).

To compare the clustering accuracy by the Yau-Hausdorff method and the feature vector method [3], we construct the UPGMA phylogentic trees of 18 species barcoding DNA data sets using the Yau-Hausdorff method and the feature vector method, the results are shown in Figs 3 and 4, respectively. Fig 3 manifests that the species with the same genus are grouped together accurately by Yau-Hausdorff method. The result is consistent with the known biological classification, by which the 18 species belong to 9 genera and each genus contains two species. The hierarchical tree in Fig 4 shows that Eunicida sp BOLDACJ5892 is gathered with genus Capitellida instead of Eunicida sp BOLDACJ9615 although they belong to the same genus, while our method clearly clusters the 18 species into 9 genera.

The results acquired from Yau-Hausdorff method is better than that from feature vector method. The feature vector does not preserve the information contained in a graphical representation because the feature vector does not contain enough information to reconstruct the curve from the vector, and thus cannot fully represent the distribution of nucleotides in a DNA sequence. Furthermore, our method includes translations and rotations for the best match of graphical curves. Thus our method offers a natural and accurate comparison of DNA sequences through graphical representations.

#### H1N1 virus analysis

We perform test on the Yau-Hausdorff method on H1N1 virus. The pandemic in 2009 was a new strain of swine-origin influenza virus(S-OIV). We analyze the polymerase PB2 segment of S-OIV (swine-origin influenza virus) 2009 as well as avian and triple reassortment swine viruses. Many researchers have conducted comprehensive computational searches to determine the origin of S-OIV. Previous study [16] indicated that the PB2 segment of S-OIV, avian, and triple reassortment swine viruses share a similar evolutionary history.

**Fig 5:**
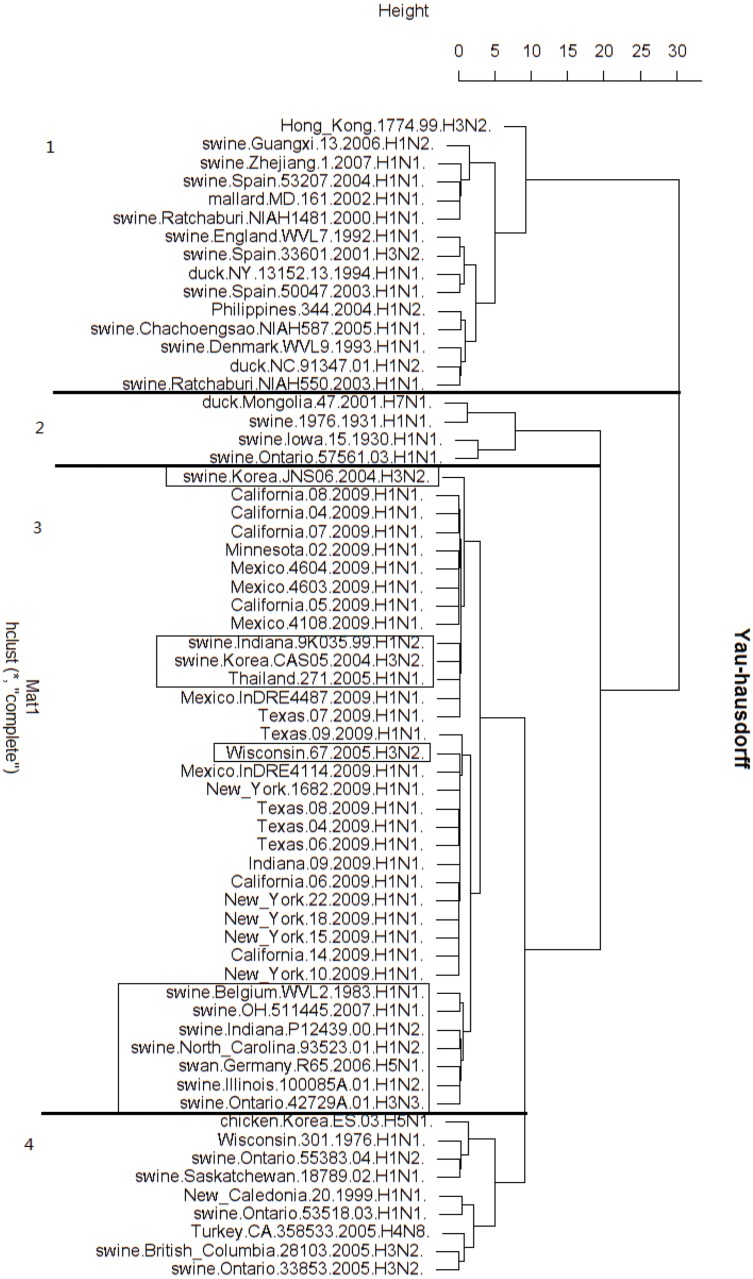
Hierarchical tree of H1N1 virus sequences (Yau-Hausdorff method).

We construct the phylogentic tree from the Yau-Hausdorff distance between the 63 virus genes (Fig 5). The hierarchical tree in Fig 5 contains four branches. The third and the fourth clusters are the nearest, and their union is juxtaposed with the second cluster, while the first cluster is the farthest from the other three. What we examine here is S-OIV 2009 PB2 genes, which are all placed into the third cluster. On the other hand, the third group contains some Swine H3N2 and H1N2 triple reassortment viruses. These clustering results agree with previous study by Kingsford [16]. The fourth group is the closest cluster to the third group. This group consists of human H1N1, triple reassortment H3N2, H2N1, chicken, and turkey virus. It was pointed out by Kingsford’s paper [16] that the PB2 gene of avian virus, reassortment H2N1, H3N2 and the S-OIV 2009 share evolutionary. The farthest group from S-OIV 2009 is the first group. This group is composed mainly of H1N1 that has been circulating in swine populations in Europe and Asia for decades. Again, these results coincide with Kingsford’s conclusion [16] which claimed that the Eurasian H1N1 has a different phylogenetic origin than the 2009 outbreak strain sequences.

#### Influenza virus gene analysis

**Fig 6:**
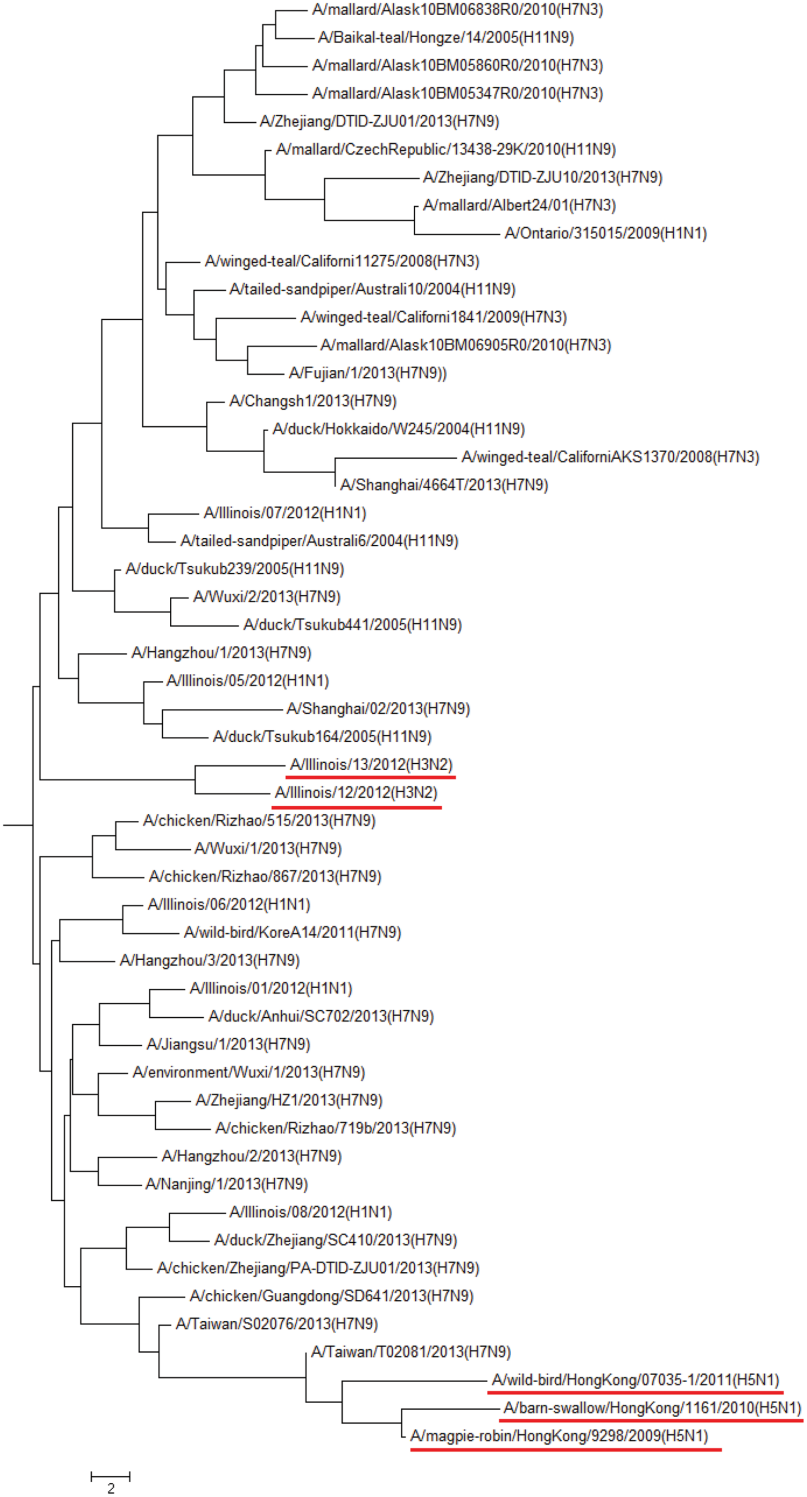
Hierarchical tree of the influenza virus NA genes(Yau-Hausdorff method).

In the last example of DNA data set, we test the influenza virus neuraminidase (NA) gene since sequence alignment does not work well for the influenza virus NA genes. We applied our method to this data set which contains 52 sequences and got the phylogenetic tree in Fig 6. The result obtained here is mainly consistent with the known biological classification. The top of this figure includes some mallard, Zhejiang and winged-teal influenza as a group. In the middle part of this figure, the Illinois influenza are gathered here. The middle and lower parts are mainly composed of H7N9 viruses, and Hong Kong influenza are clustered at the bottom of this figure. Compared with the sequence alignment method which is time consuming and not effective for the influenza virus genes, this example shows that the Yau-Hausdorff distance method works better than sequence alignment method.

### Protein sequence comparison

To assess our method on characterization of the protein space, we apply the Yau-Hausdorff method to classify the protein kinase C (PKC) family and the *β*-globin family.

In addition to hierarchical tree, we employ the natural graph to represent the classifications of protein families [17]. In the natural graph, if there is a level-1 directed edge from protein A to protein B, it means B is the closest to A among the entire family, thus they are categorized into the same level-1 cluster. Similarly a level-2 directed edge means that one level-1 cluster is the closest to another level-1 cluster. The distance between two graphs is defined as the minimum distance between any protein in one graph and any element in the other. The length of an edge that connects two proteins is proportional to the Yau-Hausdorff distance between them. A shorter line indicates a higher level of similarity between two proteins. The natural graph allows us to view the relationship between proteins and subfamilies in an intuitive way.

#### PKC family analysis

**Fig 7:**
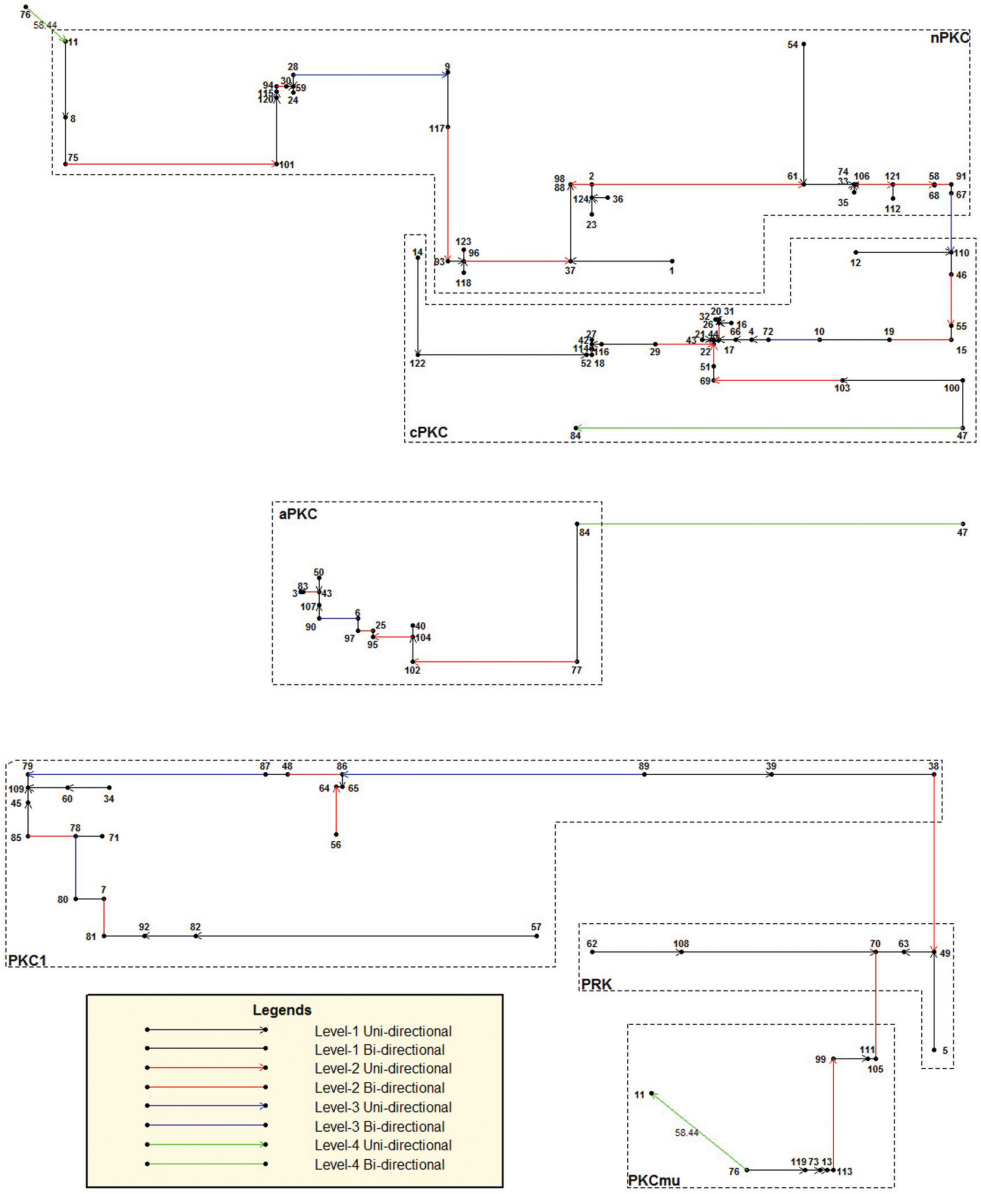
Natural graph the PKC family(Yau-Hausdorff method).

The protein kinase C family is a large group of enzymes regulating the Ca2+-dependent pathways in cells [18]. PKC is classified into six subfamilies: cPKC, nPKC, aPKC, PKC*μ*, PKC1 and PRK. We compute distance matrix from the Yau-Hausdorff distance of each pair of the 124 protein sequences in the PKC family. The natural graph constructed from the distance matrix is shown in Fig 7.

The graph shows that Yau-Hausdorff method classifies the 124 PKC sequences into three level-3 clusters with one uni-directional edge and one bi-directional edge. The first cluster contains all two typical subfamilies: cPKC (conventional PKC) and nPKC (novel PKC). The second cluster contains all aPKC sequences. One bi-directional edge connects it to the first cluster, indicating that among all PKC subfamilies, aPKC has the highest similarity with nPKC and cPKC. This matches what would be expected from their biology. The third cluster contains all the controversial PKC subfamilies. PKC*μ* is considered to be PKD actually; PKC1 are found on fungi; PRK (PKC-related kinase)are like PKC1 but they are found on animals [17]. We observe from the graph that this cluster is far from the first two. Actually the graph tells us that the minimum distance between the aPKC subfamily and the subfamilies cPKC and nPKC is 58.44, which is also the longest edge in the graph.

The Yau-Hausdorff method classifies the PKC family accurately except that PKC No. 84, which is a cPKC, is clustered into the cluster containing all aPKCs. This observation coincides with the result given by the natural vector method on the same dataset [17]. That article ascribes this abnormality yet undiscovered cPKCs that should appear between PKC No.84 and the cPKC subfamily, making PKC No.77 the closest to No.84. Our result obtained from the Yau-Hausdorff method verifies this prediction.

Examining the result more carefully we find that our clustering outcome is more accurate than that of the natural vector method. Firstly, in the results of the natural vector method, the nPKC subfamily is divided into two parts by two cPKC sequences, while the Yau-Hausdorff method clusters the PKC family into complete subfamilies. Second, the natural vector method cuts the PRK subfamily into two parts, while all pRKs are clustered into the same cluster, i.e., the same level-1 branch. That shows the Yau-Hausdorff method characterizes the distance between proteins in a way that is closer to the actual nature of the proteins than the natural vector method does, resulting in a more accurate classification.

#### *β*-globin analysis

**Fig 8:**
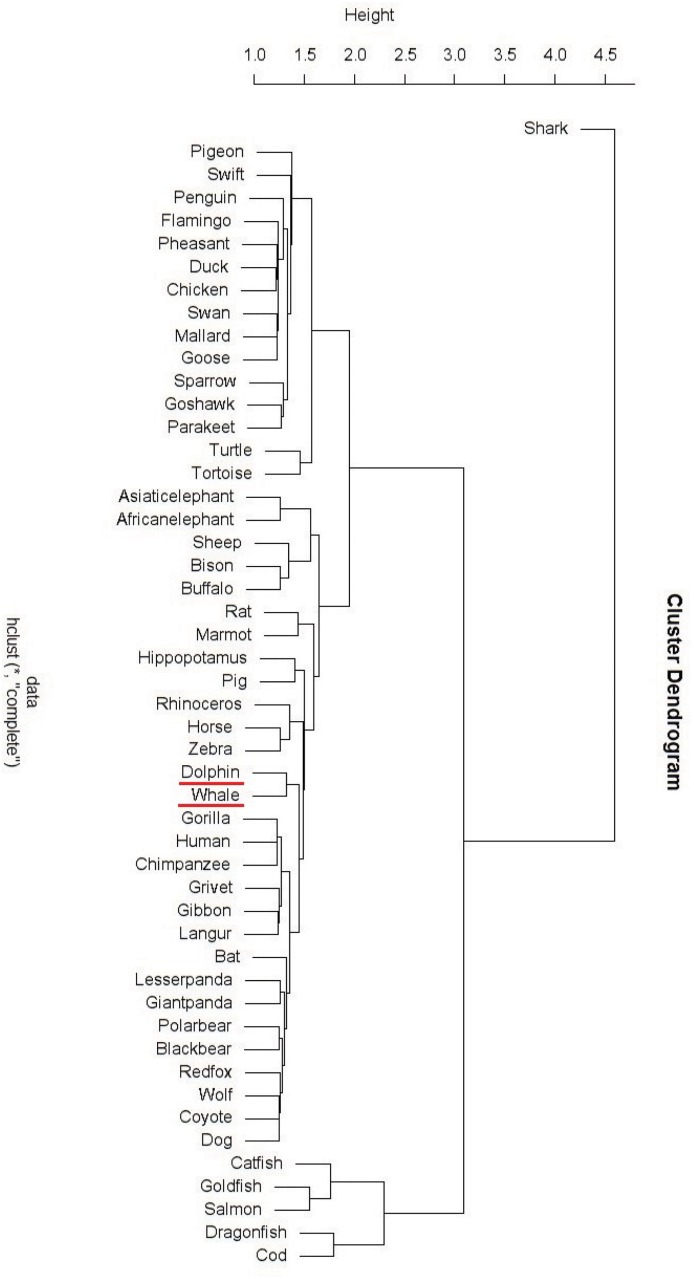
Hierarchical tree of 50 β-globin sequences (Yau-Hausdorff method).

**Fig 9:**
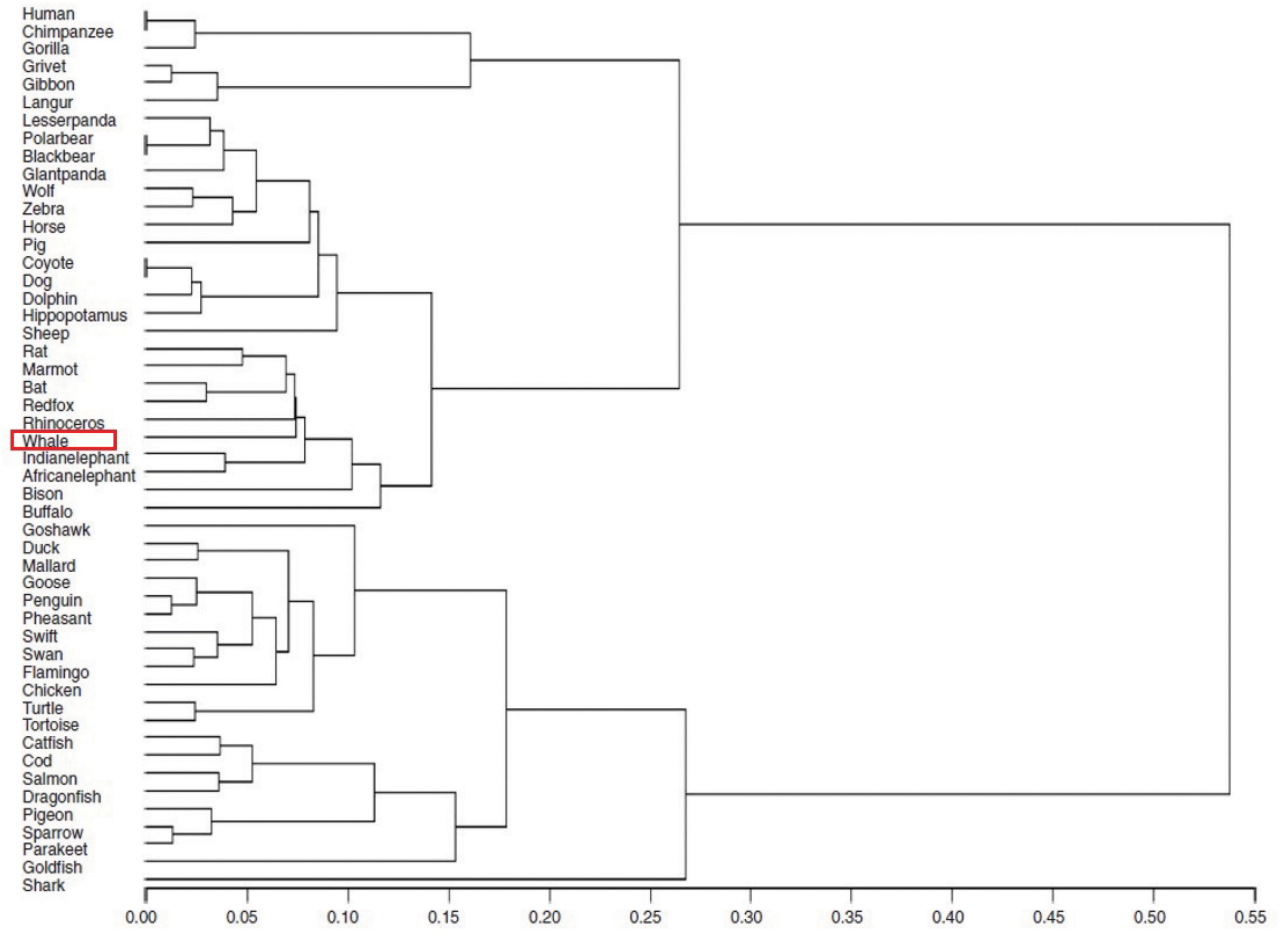
Hierarchical tree of 50 β-globin sequences (Moment vector method).

We perform phylogenetic analysis of *β*-globin from 50 species by the Yau-Hausdorff method (Fig 8) and the moment vector method (Fig 9). [2].

The hierarchical tree in Fig 9 shows that Yau-Hausdorff method categorizes the 50 *β*-globin sequences better than moment vector method. Even though the moment vector method divides the data-set into three complete parts: mammals, birds and fish, it is not as accurate as the Yau-Hausdorff clustering in regard to the classification into subfamilies of mammals. The moment vector approach leaves the subfamilies bovidae, artiodactyla, perissodactyl, whale and canidae intermingled in the final results, while our method clearly clusters the 29 mammals into elephants, bovidae, rodents, artiodactyla, perissodactyl, whales, primates, bears and canidae from top to bottom. This result is in strong agreement with biological systematics, showing that our algorithm accurately clusters the 50 species. We conclude that Yau-Hausdorff method gives a more accurate result than the moment vector method.

**Fig 10:**
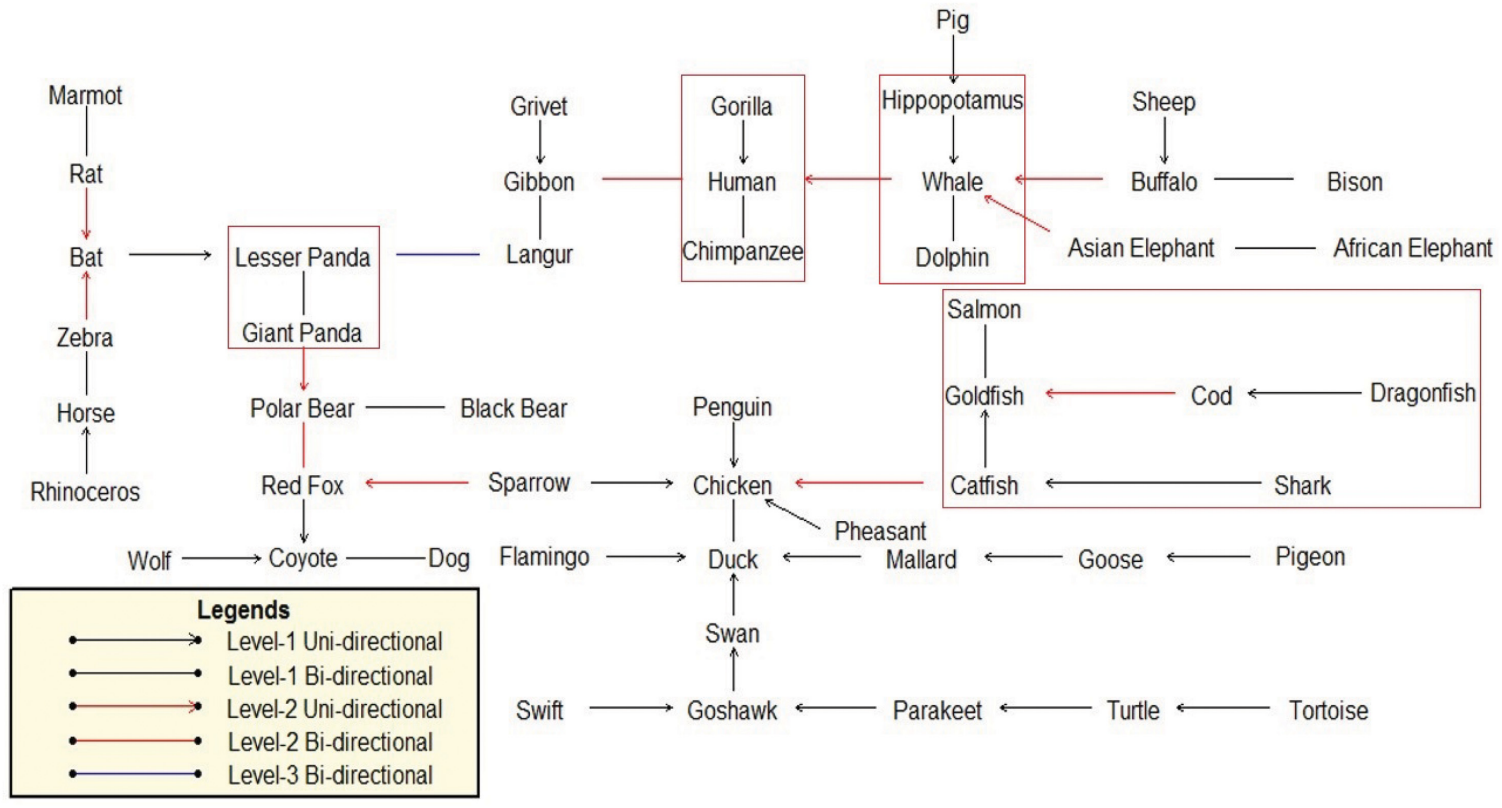
Natural graph of 50 β-globin sequences(Yau-Hausdorff method).

The only outlier in our result is that Shark is clustered to a single cluster instead of being group with other fish. Looking into the original data we find that the *β*-globin sequence of shark is the only one in the data-set with length 142, while all the 49 other sequences are of length 148. This is probably the cause of this outlier, since the algorithm generating the hierarchical tree clusters the closest branches together in each iteration. The *β*-globin sequence of shark is far from all other 49 *β*-globin (verified by observing the distance matrix) because it is shorter, and consequently it becomes branch clustered in the last step of the iteration. To correct this outlier, we represent the distance matrix with the natural graph clustering as shown in Fig 10.

The natural graph in Fig 10 shows that although the edge from Shark to Catfish is the longest in the directed graph, shark is clustered into fish by a level-1 uni-directional edge, which is what we expect. This example shows that the outlier can be resolved by the natural graph, demonstrating advantage of the natural graph and accuracy of the Yau-Hausdorff method. In summary, our method provides a distance matrix that completely agrees with established biological clustering of all 50 species.

### Stability analysis

To analyze the stability of the Yau-Hausdorff method, we apply a random perturbation within 10% to the y-coordinates of each nucleotide (amino acid) in the graphical representation of DNA (or protein) sequences. We repeat the tests on both DNA and protein data-sets several times with perturbation, and observe no structural change in the hierarchical tree or the natural graphical representation. This shows that Yau-Hausdorff distance is a natural metric with high level of stability.

### Noise perturbation analysis

**Fig 11:**
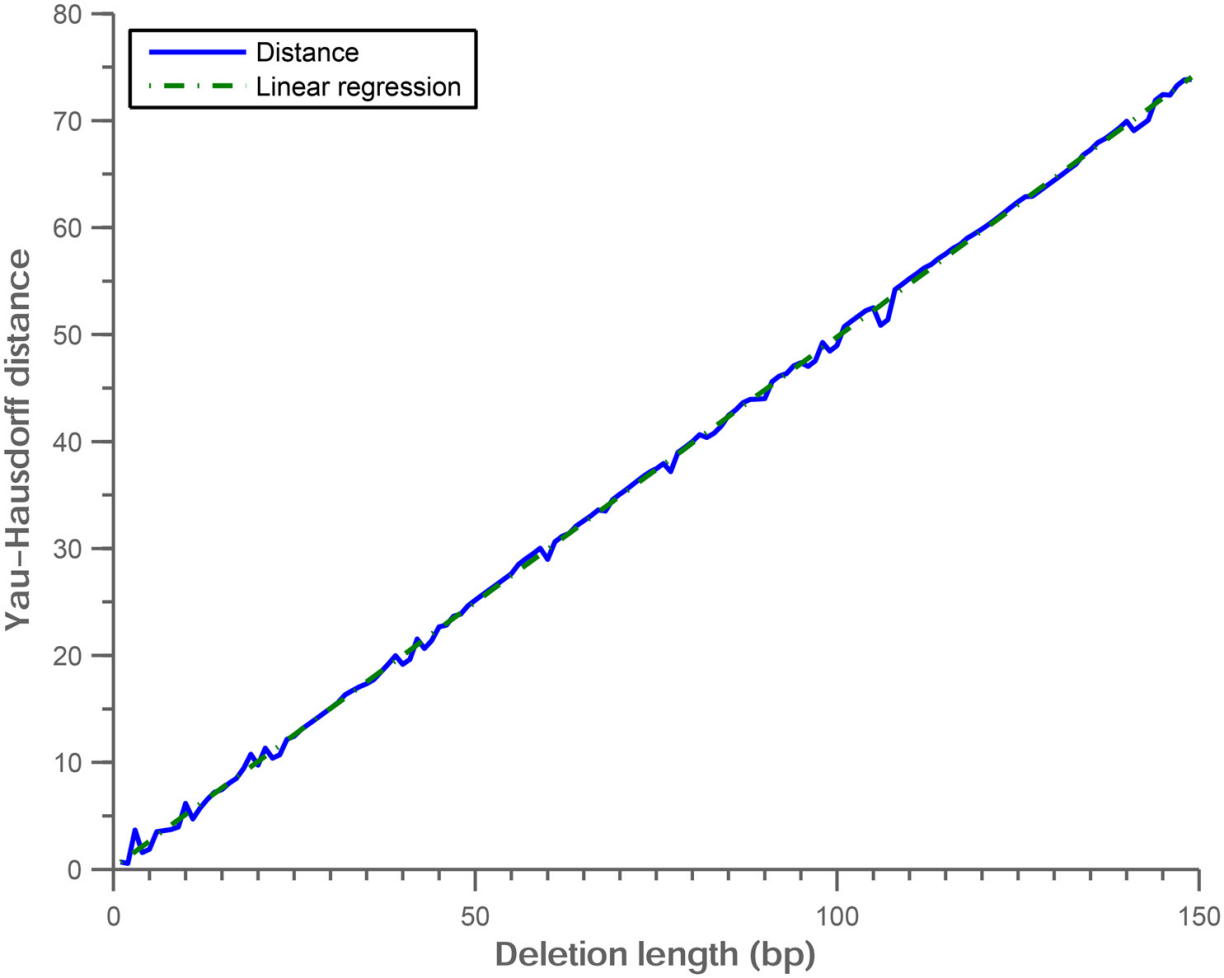
The relationship between Yau-Hausdorff distance and deletion length of sequence.

We perform the noise perturbation analysis on the Yau-Hausdorff distance. We first construct 150 deletion mutations on random positions of an intron DNA sequence of length 350 bp. The deletion mutation DNA sequences have lengths from 349 bp to 200 bp. The Yau-Hausdorff distances between each of the 150 mutation sequences and the original DNA sequence are shown in Fig 11. The result shows that the correlation of Yau-Hausdorff distance and deletion length is almost linear. This result indicates that the Yau-Hausdorff distance is robust when the complexity of the graphic representations increases or noise is introduced in the representations.

### Complexity analysis

Given two sequences with lengths *m* and *n*, the computational complexity of the Yau-Hausdorff distance between these sequence curves is *O*(*m*+*n*) times the minimum one-dimensional Hausdorff distance. For two sets of *m* and *n* points, the complexity of their one-dimensional minimum Hausdorff distance is *O*((*m*+*n*) log(*m*+*n*)) based on Li’s algorithm [12]. Therefore, the complexity of our algorithm is *O*((*m*+*n*)^2^ log(*m*+*n*)). Existing algorithms which find the minimum Hausdorff distance for point sets under Euclidean motion are highly complex, with *O*((*m*+*n*)^5^ log^2^ *mn*)) for the fastest algorithm [8]. The Yau-Hausdorff distance method significantly decreases computational complexity of finding the minimum Hausdorff distance between two-dimensional shapes.

## Discussion

In this study, the Yau-Hausdorff method provides more accurate results compared with the existing methods. Unlike previous methods that attempt to numerically characterize DNA or protein sequence graphical curves, the Yau-Hausdorff method measures the distance between sequences by directly comparing the curves via the Yau-Hausdorff distance. It avoids potential information loss caused by the transformation from graphical curves into numerical characterizations. Our approach takes translations and rotations into consideration the best match of graphical curves. It offers a more natural and accurate comparison of the sequence graphical representations. In addition, since the Yau-Hausdorff method is based on graphical representations of DNA(or protein) sequences, it also has the advantage of being intuitive. The graphical representation serves to intuitively depict the distribution of nucleotides (amino acids) in a sequence, and our method inherits that property.

The minimum Hausdorff distance of shapes is an important measure for similarity comparison and shape matching and retrievals, but computation of the Hausdorff distance in two and higher dimensions is a challenging problem. We propose this novel solution to this problem and prove that Yau-Hausdorff distance in *d*-dimensional Euclidean space ℝ^*d*^ is also a metric for *d* > 2 (proof available in S1 File). It has potential broad application prospects in measuring the similarity of 2 or 3-dimensional curves, such as shape matching, image retrieval, and comparison of 3-dimensional protein structures.

Although the Yau-Hausdorff method has these advantages, it also has some limitations. The phylogenetic tree constructed by this distance may contain uncertainties. For example, the phylogenetic tree in Fig 8 constructed by this distance showed that shark is not grouped with other fish. Another problem is that the computation complexity is high if the sequences contain millions of nucleotides. We will continue to solve the limitations and improve the complexity of the algorithm.

## Conclusion

This article proposes a new approach for comparing DNA sequences and protein sequences. We introduce two fundamental tools of our method: the graphical representation of DNA(or protein) sequences and the Yau-Hausdorff distance. We then define the distance between two sequences using the Yau-Hausdorff distance between the two-dimensional graphical representations of the sequences. Given a family of DNA(or protein), we use this approach to calculate the distance between each pair of sequences, getting a distance matrix that contains information about the family structure.

In the tests on both DNA and protein data-sets, we use hierarchical tree and natural graphical representation to analyze the distance matrix. Based on different kinds of datasets, our results show that the Yau-Hausdorff method gives the most accurate clustering compared with several other approaches including natural vector method, feature vector method, moment vector method and sequence alignment method on both DNA and protein families. In addition, we perform our test repeatedly with perturbation. The perturbation does not affect our results. We conclude that the Yau-Hausdorff method is a natural, accurate and stable approach for comparing DNA sequences and protein sequences, and has general applications in shape and image matching.

## Supporting Information

S1 File Proof that Yau-Hausdorff distance is a metric, and is always less than or equal to the minimum two-dimensional Hausdorff distance. (PDF)

S2 File GenBank access numbers of DNA and protein sequences used in this study. (XLSX)

## References

[1] Altschul SF, Gish W, Miller W, Myers EW, Lipman DJ. Basic local alignment search tool. Journal of molecular biology. 1990;215(3):403–410. 2231712 10.1016/S0022-2836(05)80360-2

[2] Yau SST, Yu C, He R. A protein map and its application. DNA and cell biology. 2008;27(5):241–250. 10.1089/dna.2007.0676 18348704

[3] Huang G, Zhou H, Li Y, Xu L. Alignment-free comparison of genome sequences by a new numerical characterization. Journal of theoretical biology. 2011;281(1):107–112. 10.1016/j.jtbi.2011.04.003 21536050

[4] Liu B, Liu F, Fang L, Wang X, Chou KC. repDNA: a Python package to generate various modes of feature vectors for DNA sequences by incorporating user-defined physicochemical properties and sequence-order effects. Bioinformatics. 2015;31(8):1307–1309. 10.1093/bioinformatics/btu820 25504848

[5] Zou Q, Hu Q, Guo M, Wang G. HAlign: Fast multiple similar DNA/RNA sequence alignment based on the centre star strategy. Bioinformatics. 2015;p. btv177. 10.1093/bioinformatics/btv177 25812743

[6] Yau SST, Wang J, Niknejad A, Lu C, Jin N, Ho YK. DNA sequence representation without degeneracy. Nucleic acids research. 2003;31(12):3078–3080. 10.1093/nar/gkg432 12799435 PMC162336

[7] Huttenlocher DP, Klanderman GA, Rucklidge WJ. Comparing images using the Hausdorff distance. Pattern Analysis and Machine Intelligence, IEEE Transactions on. 1993;15(9):850–863. 10.1109/34.232073

[8] Chew LP, Goodrich MT, Huttenlocher DP, Kedem K, Kleinberg JM, Kravets D. Geometric pattern matching under Euclidean motion. Computational Geometry. 1997;7(1):113–124. 10.1016/0925-7721(95)00047-X

[9] Fauchere J, Pliska V. Hydrophobic parameters-pi of amino-acid side-chains from the partitioning of N-acetyl-amino-acid amides. European Journal of Medicinal Chemistry. 1983;18(4):369–375.

[10] Huttenlocher DP, Kedem K, Kleinberg JM. On dynamic Voronoi diagrams and the minimum Hausdorff distance for point sets under Euclidean motion in the plane. In: Proceedings of the eighth annual symposium on Computational geometry. ACM; 1992. p. 110–119.

[11] Rote G. Computing the minimum Hausdorff distance between two point sets on a line under translation. Information Processing Letters. 1991;38(3):123–127. 10.1016/0020-0190(91)90233-8

[12] Li B, Shen Y, Li B. A new algorithm for computing the minimum Hausdorff distance between two point sets on a line under translation. Information Processing Letters. 2008;106(2):52–58. 10.1016/j.ipl.2007.10.003

[13] Sourdis J, Krimbas C. Accuracy of phylogenetic trees estimated from DNA sequence data. Molecular biology and evolution. 1987;4(2):159–166. 3447006 10.1093/oxfordjournals.molbev.a040432

[14] Hebert PD, Cywinska A, Ball SL, et al Biological identifications through DNA barcodes. Proceedings of the Royal Society of London Series B: Biological Sciences. 2003;270(1512):313–321. 10.1098/rspb.2002.2218 12614582 PMC1691236

[15] Deng M, Yu C, Liang Q, He RL, Yau SST. A novel method of characterizing genetic sequences: genome space with biological distance and applications. PloS one. 2011;6(3):e17293 10.1371/journal.pone.0017293 21399690 PMC3047556

[16] Kingsford C, Nagarajan N, Salzberg SL. 2009 Swine-origin influenza A (H1N1) resembles previous influenza isolates. Plos one. 2009;4(7):e6402 10.1371/journal.pone.0006402 19636415 PMC2712239

[17] Yu C, Deng M, Cheng SY, Yau SC, He RL, Yau SST. Protein space: a natural method for realizing the nature of protein universe. Journal of theoretical biology. 2013;318:197–204. 10.1016/j.jtbi.2012.11.005 23154188

[18] Nishizuka Y. Studies and perspectives of protein kinase C. Science. 1986;233(4761):305–312. 10.1126/science.3014651 3014651

