# Supplementary material for: Two Dimensional Yau-Hausdorff Distance with Applications on Comparison of DNA and Protein Sequences
Source: PLoS One. 2015 Sep 18;10(9):e0136577. doi: 10.1371/journal.pone.0136577 (PMC4575136; doi:10.1371/journal.pone.0136577)
Supplement: S1 File — (PDF) [file pone.0136577.s001.pdf]

1 Proof that Yau-Hausdorff distance is a metric

1.1 Lemma 1:

Let A and B be two sets of finite points in $\mathbb{R}^d$, $d(a,b) = |a-b|$ is the Euclidean distance. For $a \in A$, we define $d(a,B) = \min_{b\in B} d(a,b)$. Similarly, we define $d(b,A) = \min_{a\in A} d(b,a)$. Then define $d(A,B) = \max_{a\in A} d(a,B)$, $d(B,A) = \max_{b\in B}$ d(b,A) and $h(A,B) = \max\{d(A,B), d(B,A)\}$. Then h is a metric.
Proof:

1. Obviously $h \geq 0$.

   If $h(A,B) = 0$, then $d(A,B) = d(B,A) = 0$. $\max_{a\in A} d(a,B) = 0$, implies for each $a \in A$ $d(a,B)$=0. We have $\min_{b\in B} d(a,b) = 0$ for any $a \in A$. Because B is a finite set, we can find $b \in B$, s.t. $b = a$.

   This gives us $A \subset B$, similarly we have $B \subset A$. Hence $A = B$.

   On the other hand if $A = B$, we have $h(A,B) = 0$ from definition, so $h(A,B) = 0$ if and only if $A = B$.

2. $h(A,B) = \max\{d(A,B), d(B,A)\} = \max\{d(B,A), d(A,B)\} = h(B,A)$.

3. We take three sets finite point sets A, B, C in $\mathbb{R}^d$ and show that $h(A,B) \leq h(A,C) + h(C,B)$, i.e.

   $$\max\{d(A,B), d(B,A)\} \leq \max\{d(A,C), d(C,A)\} + \max\{d(C,B), d(B,C)\} \tag{1}$$

   First we show that

   $$d(a,B) \leq d(a,C) + d(C,B) \tag{2}$$

   for each $a \in A$.

Assume

$$d(a,C) = \min_{c \in C} d(a,c) = d(a,c_0), c_0 \in C \tag{3}$$

$$d(c_0,B) = \min_{b \in B} d(c_0,b) = d(c_0,b_0), b_0 \in B \tag{4}$$

It follows that

$$\begin{aligned} d(a,B) \leq & d(a,b_0) && (5) \\ \leq & d(a,c_0) + d(c_0,b_0) && (6) \\ = & d(a,C) + d(c_0,B) && (7) \\ \leq & d(a,C) + d(C,B) && (8) \end{aligned}$$

and equation (2) holds. Hence

$$\begin{aligned} d(a,B) \leq & d(a,C) + d(C,B) && (9) \\ \leq & d(A,C) + d(C,B) && (10) \\ \leq & \max\{d(A,C), d(C,A)\} + \max\{d(C,B), d(B,C)\} && (11) \\ = & h(A,C) + h(C,B) && (12) \end{aligned}$$

for each fixed $a \in A$.

Take the maximum of the left hand of this inequality,

$$d(A,B) = \max_{a \in A} d(a,B) \leq h(A,C) + h(C,B) \tag{13}$$

Similarly we can get

$$d(B,A) \leq h(A,C) + h(C,B) \tag{14}$$

$$h(A,B) = \max\{d(A,B), d(B,A)\} \leq h(A,C) + h(C,B) \tag{15}$$

The triangle inequality holds.

We have proven that h is a metric.

1.2 Lemma 2:

Let A and B be two sets of finite points in $\mathbb{R}^d$. For a translation vector $t \in \mathbb{R}^d$, we define $A + t = \{a + t | a \in A\}$. For a rotation θ, we define A^θ to be the set A rotated around the origin by θ. Let $H^d(A, B) = \inf_{t,\theta} h(A^\theta + t, B)$, then H^d is a metric, and is called minimum d-dimensional Hausdorff metric.
Proof:

1. $$H^d(A, B) = \inf_{t,\theta} h(A^\theta + t, B) \geq 0 \tag{16}$$

   If $H^d(A, B) = 0$, then we can find t_0 and θ_0, such that $h(A^{\theta_0} + t_0, B) = 0$. From Lemma 1 we have $A^{\theta_0} + t_0 = B$ in $\mathbb{R}^d$, so $A \stackrel{\triangle}{=\!=} B$. (Here $A \stackrel{\triangle}{=\!=} B$ means that A and B are of the same shape, i.e. we can find translation t and rotation θ, such that $A^\theta + t = B$).

   On the other hand, if $A \stackrel{\triangle}{=\!=} B$, then we can find t_0 and θ_0, s.t. $A^{\theta_0} + t_0 = B$.

   Then $h(A^{\theta_0} + t_0, B) = 0$ and $H^d(A, B) = \inf_{t,\theta} h(A^\theta + t, B) = 0$.

   $H^d(A, B) = 0$ if and only if $A \stackrel{\triangle}{=\!=} B$.

2.

$$H^d(A, B) \tag{17}$$
$$= \inf_{t,\theta} h(A^\theta + t, B) \tag{18}$$
$$= \inf_{t,\theta} h(A, (B - t)^{-\theta}) \tag{19}$$
$$= \inf_{t,\theta} h((B - t)^{-\theta}, A) \tag{20}$$
$$= \inf_{t,\theta} h(B^{-\theta} - t, A) \tag{21}$$
$$= \inf_{t',\theta'} h(B^{\theta'} + t', A) \tag{22}$$
$$= H^d(B, A) \tag{23}$$

3. Take three finite point sets A, B, C in $\mathbb{R}^d$ and show that $H^d(A, B) \leq H^d(A, C) + H^d(B, C)$. This is equivalent to

$$\inf_{t,\theta} h(A^\theta + t, B) \leq \inf_{t,\theta} h(A^\theta + t, C) + \inf_{t,\theta} h(B^\theta + t, C) \tag{24}$$

Since the rotation group is compact and we only need to consider the translation in a compact region, we can find θ_1, t_1, θ_2, t_2, s.t.

$$h(A^{\theta_1} + t_1, C) = \inf_{t,\theta} h(A^\theta + t, C) \tag{25}$$
$$h(B^{\theta_2} + t_2, C) = \inf_{t,\theta} h(B^\theta + t, C) \tag{26}$$

That gives us

$$H^d(A,C) + H^d(B,C) \tag{27}$$

$$= \inf_{t,\theta} h(A^\theta + t, C) + \inf_{t,\theta} h(B^\theta + t, C) \tag{28}$$

$$= h(A^{\theta_1} + t_1, C) + h(B^{\theta_2} + t_2, C) \tag{29}$$

$$\geq h(A^{\theta_1} + t_1, B^{\theta_2} + t_2) \tag{30}$$

$$= h(A^{\theta_1} + t_1 - t_2, B^{\theta_2}) \tag{31}$$

$$= h((A^{\theta_1} + t_1 - t_2)^{-\theta_2}, B) \tag{32}$$

$$= h(A^{\theta_1 - \theta_2} + t_1 - t_2, B) \tag{33}$$

$$\geq \inf_{t,\theta} h(A^\theta + t, B) \tag{34}$$

$$= H^d(A,B) \tag{35}$$

The triangle inequality holds.

We have proven that H^d is a metric.

1.3 Theorem:

Let A and B be two point sets of finite points in $\mathbb{R}^2$. For a rotation θ, we define $P_x(A^\theta)$ to be the x-axis projection of A^θ.

$$\begin{aligned} D(A,B) = \max\{ &\sup_\theta \inf_\varphi H^1(P_x(A^\theta), P_x(B^\varphi)), \\ &\sup_\varphi \inf_\theta H^1(P_x(A^\theta), P_x(B^\varphi))\} \end{aligned} \tag{36}$$

Here H^1 is the minimum one-dimensional Hausdorff distance,

$$H^1(A,B) = \inf_{t\in\mathbb{R}} \max\{\max_{a\in A+t} \min_{b\in B} |a-b|, \max_{b\in B} \min_{a\in A+t} |b-a|\} \tag{37}$$

then D is a metric.

Proof:

1.
$$D(A,B) = \max\{\sup_{\theta}\inf_{\varphi} H^1(P_x(A^\theta), P_x(B^\varphi)),\\ \sup_{\varphi}\inf_{\theta} H^1(P_x(A^\theta), P_x(B^\varphi))\} \tag{38}$$

$$D(B,A) = \max\{\sup_{\theta}\inf_{\varphi} H^1(P_x(B^\theta), P_x(A^\varphi)),\\ \sup_{\varphi}\inf_{\theta} H^1(P_x(B^\theta), P_x(A^\varphi))\} \tag{39}$$

Since $H^1(A,B) = H^1(B,A)$, we have

$$\sup_{\theta}\inf_{\varphi} H^1(P_x(A^\theta), P_x(B^\varphi)) = \sup_{\varphi}\inf_{\theta} H^1(P_x(B^\theta), P_x(A^\varphi))\\ \sup_{\varphi}\inf_{\theta} H^1(P_x(A^\theta), P_x(B^\varphi)) = \sup_{\theta}\inf_{\varphi} H^1(P_x(B^\theta), P_x(A^\varphi)) \tag{40}$$

which gives us D(A,B)=D(B,A).

2. We take three sets A,B,C of finite points in $\mathbb{R}^2$ and show that $D(A,B) \leq D(A,C) + D(C,B)$. First we prove that

$$\inf_{\varphi} H^1(P_x(A^{\theta_0}), P_x(B^\varphi)) \leq D(A,C) + D(C,B) \tag{41}$$

for each fixed θ_0. Assume α_0 is a rotation, s.t.

$$H^1(P_x(A^{\theta_0}), P_x(C^{\alpha_0})) = \inf_{\alpha} H^1(P_x(A^{\theta_0}), P_x(C^\alpha)) \tag{42}$$

φ_0 is a rotation, s.t.

$$H^1(P_x(C^{\alpha_0}), P_x(B^{\varphi_0})) = \inf_{\varphi} H^1(P_x(C^{\alpha_0}), P_x(B^\varphi)) \tag{43}$$

So

$$\inf_{\varphi} H^1(P_x(A^{\theta_0}), P_x(B^{\varphi})) \tag{44}$$

$$\leq H^1(P_x(A^{\theta_0}), P_x(B^{\varphi_0})) \tag{45}$$

$$\leq H^1(P_x(A^{\theta_0}), P_x(C^{\alpha_0})) + H^1(P_x(C^{\alpha_0}), P_x(B^{\varphi_0})) \tag{46}$$

$$= \inf_{\alpha} H^1(P_x(A^{\theta_0}), P_x(C^{\alpha})) + \inf_{\varphi} H^1(P_x(C^{\alpha_0}), P_x(B^{\varphi})) \tag{47}$$

$$\leq \sup_{\theta} \inf_{\alpha} H^1(P_x(A^{\theta}), P_x(C^{\alpha})) + \sup_{\alpha} \inf_{\varphi} H^1(P_x(C^{\alpha}), P_x(B^{\varphi})) \tag{48}$$

$$\leq D(A, C) + D(C, B) \tag{49}$$

for each fixed rotation θ_0.

We take the maximum of all rotation θ in the left hand, and we get

$$\sup_{\theta} \inf_{\varphi} H^1(P_x(A^{\theta}), P_x(B^{\varphi})) \leq D(A, C) + D(C, B) \tag{50}$$

Similarly, we can get

$$\sup_{\varphi} \inf_{\theta} H^1(P_x(A^{\theta}), P_x(B^{\varphi})) \leq D(A, C) + D(C, B) \tag{51}$$

So

$$D(A, B) = \max\{\sup_{\theta} \inf_{\varphi} H^1(P_x(A^{\theta}), P_x(B^{\varphi})), \sup_{\varphi} \inf_{\theta} H^1(P_x(A^{\theta}), P_x(B^{\varphi}))\} \tag{52}$$

$$\leq D(A, C) + D(C, B) \tag{53}$$

The triangle inequality holds.

3. Obviously $D(A, B) \geq 0$ for any two point sets A,B.

   We need to prove that $A \overset{\triangle}{=\!=} B$ if and only if $D(A, B) = 0$.

   If $A \overset{\triangle}{=\!=} B$, then $D(A, B) = 0$.

   Conversely, if $D(A, B) = 0$, we need to show that $A \overset{\triangle}{=\!=} B$.

Assume that there are m points in set A and n points in set B. We assume that $m > n$.

We can find a rotation θ_0, s.t. the number of points in $P_x(A^{\theta_0})$ has m different points, but the number of points in $P_x(B^{\varphi})$ is no more than n, so

$$P_x(A^{\theta_0}) \neq P_x(B^{\varphi}) \tag{54}$$

$$\Longrightarrow \inf_{\varphi} H^1(P_x(A^{\theta}), P_x(B^{\varphi})) > 0 \tag{55}$$

$$\Longrightarrow D(A, B) > 0 \tag{56}$$

Contradiction! So we must have $m \leq n$. Similarly we can get $n \leq m$. So $m = n$. The number of points of the two sets must be the same.

We consider a new question. If we know all the x-axis projections of set A with different rotation θ, can we reconstruct set A in the x,y-plane? This question is equivalent to the original question because all the projection of set A and set B are the same if $D(A, B) = 0$, and we are about to show that the answer of this new question is yes.

First we consider a simple situation. There are only three different points in set A. Without loss of generality we fix a point at the origin. Then we rotate the set A three times so that each time a line that connects two points of A parallels the x-axis. So we can know the distance of any two points in set A from the information of projections, then the shape of set A is fixed.

If there are n different points in set A, again we fix a point at the origin O. Similarly we can determine the shape of the triangle $\triangle OA_1A_2$ with three rotations.

For the next point A_3, we can know the distance between A_3,O, the distance between A_3,A_1 and the distance between A_3,A_2 by three rotations. So the location of A_3 is fixed. The other points are fixed in the same way.

For each point, we need three other rotations. So with $3+3(n-3) = 3n-6$ rotations, the shape of A is fixed.

It means that we can reconstruct the set A in a plane from the information of $P_x(A^\theta)$ for all θ. If $D(A,B) = 0$, the projections of A and B with all the rotations are the same. $A \overset{\triangle}{=\!=} B$.

With symmetry, triangle inequality, non-negativity and identity of indiscernibles as shown above, we have proven that D is a metric. Q.E.D.

Remark: This theorem has a more general version. $D(A,B)$ defined in Euclidean space $\mathbb{R}^d$ is a metric, for all $d \geq 2$.
Proof: Symmetry, triangle inequality an non-negativity can be proven the same way above. We only need to prove identity of indiscernibles.
Again, we only need to show that we can reconstruct set A up to rigid motion in $\mathbb{R}^d$ with all the x-axis projections of A with different rotation θ. For d=2, we have shown that $3n-6$ rotations is enough to reconstruct A. There is a similar formula for arbitrary d. Once we reconstruct A in $\mathbb{R}^d$ with all the x-axis projections of A with different rotation θ, $D(A,B) = 0$ gives us $A \overset{\triangle}{=\!=} B$.
We have proven that D is a metric.

2 Proof that $H^2(A,B) \geq D(A,B)$

2.1 Lemma

Let $A = \{a_1, a_2, ..., a_n\} \subset \mathbb{R}^2$, $B = \{b_1, b_2, ..., b_m\} \subset \mathbb{R}^2$. Let

$$d(A,B) = \max_{1\leq i\leq n} \min_{1\leq j\leq m} d(a_i, b_j) \tag{57}$$

$$d(B,A) = \max_{1\leq j\leq m} \min_{1\leq i\leq n} d(b_j, a_i) \tag{58}$$

$$h(A,B) = \max\{d(A,B), d(B,A)\} \tag{59}$$

then

$$h(A^\theta, B^\varphi) \geq H^1(P_x(A^\theta), P_x(B^\varphi)) \tag{60}$$

for any rotation θ and φ. Here H^1 is the minimum one-dimensional Hausdorff distance.

Proof: Assume $A^\theta = \{a_{1\theta}, a_{2\theta}, ..., a_{n\theta}\}$, $B^\varphi = \{b_{1\varphi}, b_{2\varphi}, ..., b_{m\varphi}\}$, $P_x(A^\theta) = \{x_{1\theta}, x_{2\theta}, ..., x_{n\theta}\}$, $P_x(B^\varphi) = \{y_{1\varphi}, y_{2\varphi}, ..., y_{m\varphi}\}$. $x_{i\theta}$ is the x-projection of $a_{i\theta}, 1 \leq i \leq n$ and $y_{j\varphi}$ is the x-projection of $b_{j\varphi}, 1 \leq j \leq m$.

$d(a_{i\theta}, b_{j\varphi}) \geq d(x_{i\theta}, y_{j\varphi})$ for any i,j. Take the minimum of $j = 1, 2, ..., m$ in this inequality, and we get

$$\min_{1 \leq j \leq m} d(a_{i\theta}, b_{j\varphi}) \geq \min_{1 \leq j \leq m} d(x_{i\theta}, y_{j\varphi}) \tag{61}$$

Take the max of $i = 1, 2, ..., n$ in this inequality, and we get

$$\max_{1 \leq i \leq n} \min_{1 \leq j \leq m} d(a_{i\theta}, b_{j\varphi}) \geq \max_{1 \leq i \leq n} \min_{1 \leq j \leq m} d(x_{i\theta}, y_{j\varphi}) \tag{62}$$

This means

$$d(A^\theta, B^\varphi) \geq d(P_x(A^\theta), P_x(B^\varphi)) \tag{63}$$

Similarly we have

$$d(B^\varphi, A^\theta) \geq d(P_x(B^\varphi), P_x(A^\theta)) \tag{64}$$

$$h(A^\theta, B^\varphi) \tag{65}$$

$$= \max\{d(A^\theta, B^\varphi), d(B^\varphi, A^\theta)\} \tag{66}$$

$$\geq \max\{d(P_x(A^\theta), P_x(B^\varphi)), d(P_x(B^\varphi), P_x(A^\theta))\} \tag{67}$$

$$= h(P_x(A^\theta), P_x(B^\varphi)) \tag{68}$$

$$\geq \inf_{t \in \mathbb{R}} h(P_x(A^\theta) + t, P_x(B^\varphi)) \tag{69}$$

$$= H^1(P_x(A^\theta), P_x(B^\varphi)) \tag{70}$$

Q.E.D.

2.2 Theorem

Let $A = \{a_1, a_2, ..., a_n\} \subset \mathbb{R}^2$, $B = \{b_1, b_2, ..., b_m\} \subset \mathbb{R}^2$. $H^2(A, B)$ is the minimum two-dimensional Hausdorff distance of A and B, i.e.

$H^2(A, B) = \inf_{t\in\mathbb{R}^2} \inf_\theta h(A^\theta + t, B)$

$D(A, B) = \max\{\sup_\theta \inf_\varphi H^1(P_x(A^\theta), P_x(B^\varphi)), \sup_\varphi \inf_\theta H^1(P_x(A^\theta), P_x(B^\varphi))\}$.

Then $H^2(A, B) \geq D(A, B)$.

Proof: Assume

$$d(A,B) = \max_{1\leq i\leq n} \min_{1\leq j\leq m} d(a_i, b_j) \tag{71}$$

$$d(B,A) = \max_{1\leq j\leq m} \min_{1\leq i\leq n} d(b_j, a_i) \tag{72}$$

$$h(A,B) = \max\{d(A,B), d(B,A)\} \tag{73}$$

$$H^2(A,B) = \inf_{t\in\mathbb{R}^2} \inf_\theta h(A^\theta + t, B) \tag{74}$$

First we prove that $h(A^{\theta_1} + t_1, B) \geq D(A, B)$ for any fixed θ_1 and t_1. We only need to show that $h(A^{\theta_1} + t_1, B) \geq \sup_\theta \inf_\varphi H^1(P_x(A^\theta), P_x(B^\varphi))$.

Fix $\theta = \theta_2$,

$$h(A^{\theta_1} + t_1, B) \tag{75}$$

$$=h(A^{\theta_1}, B - t_1) \tag{76}$$

$$=h(A, (B - t_1)^{-\theta_1}) \tag{77}$$

$$=h(A^{\theta_2}, (B - t_1)^{-\theta_1+\theta_2}) \tag{78}$$

$$=h(A^{\theta_2}, B^{-\theta_1+\theta_2} - t_1) \tag{79}$$

$$\geq H^1(P_x(A^{\theta_2}), P_x(B^{-\theta_1+\theta_2} - t_1)) \tag{80}$$

$$=H^1(P_x(A^{\theta_2}), P_x(B^{-\theta_1+\theta_2})) \tag{81}$$

$$\geq \inf_\varphi H^1(P_x(A^{\theta_2}), P_x(B^\varphi)) \tag{82}$$

Equation (80) above is from the lemma. That gives us

$$h(A^{\theta_1}+t_1,B)\geq \inf_{\varphi} H^1(P_x(A^{\theta_2}),P_x(B^{\varphi})) \tag{83}$$

for any fixed θ_2, which means

$$h(A^{\theta_1}+t_1,B)\geq \sup_{\theta}\inf_{\varphi} H^1(P_x(A^{\theta}),P_x(B^{\varphi})) \tag{84}$$

Similarly we can get

$$h(A^{\theta_1}+t_1,B)\geq \sup_{\varphi}\inf_{\theta} H^1(P_x(A^{\theta}),P_x(B^{\varphi})) \tag{85}$$

Equations (84) and (85) give us

$$h(A^{\theta_1}+t_1,B) \tag{86}$$
$$\geq \max\{\sup_{\theta}\inf_{\varphi} H^1(P_x(A^{\theta}),P_x(B^{\varphi})),\sup_{\varphi}\inf_{\theta} H^1(P_x(A^{\theta}),P_x(B^{\varphi}))\} \tag{87}$$
$$=D(A,B) \tag{88}$$

for any θ_1 and t_1. Take minimum of the left hand, and we have

$$\inf_{t\in\mathbb{R}^2}\inf_{\theta} h(A^{\theta}+t,B)\geq D(A,B) \tag{89}$$
$$\Longrightarrow H^2(A,B)\geq D(A,B) \tag{90}$$

Q.E.D.

3 A simple example

Let $A=\{(0,0),(0,1),(1,0),(1,1)\}\subset\mathbb{R}^2$,$B=\{(0,0),(0,1),(1,1)\}\subset\mathbb{R}^2$. We will show that $H^2(A,B)=\frac{1}{2}>\frac{\sqrt{5}}{10}=D(A,B)$

3.1 Compute $H^2(A,B)$

First we prove that $h(A, B^\theta + t) \geq \frac{1}{2}$ for all fixed θ and t.

Draw 4 disks of radius $\frac{1}{2}$ centered at $O(0,0), M(0,1), P(1,0), N(1,1)$. Because there are three points in $B^\theta + t$, there must be a disk that does not contain any point of $B^\theta + t$. We denote the four disks C_O, C_M, C_N, C_P and assume that there is no point of $B^\theta + t$ in C_O.

So

$$\min_{b_j \in B^\theta + t} d(O, b_j) \geq \frac{1}{2} \tag{91}$$

$$\Longrightarrow d(O, B^\theta + t) \geq \frac{1}{2} \tag{92}$$

which gives us

$$d(A, B^\theta + t) = \max_{a_i \in A} d(a_i, B^\theta + t) \geq \frac{1}{2} \tag{93}$$

$$h(A, B^\theta + t) = \max\{d(A, B^\theta + t), d(B^\theta + t, A)\} \geq \frac{1}{2} \tag{94}$$

Take minimum of the left hand of equation (94), we have

$$\inf_{t \in \mathbb{R}^2} \inf_{\theta} h(A, B^\theta + t) \geq \frac{1}{2} \tag{95}$$

We then show that $\inf_{t \in \mathbb{R}^2} \inf_\theta h(A, B^\theta + t) = \frac{1}{2}$.

Take a rigid motion from B to $B' = \{(\frac{1}{2}, 0), (\frac{3}{2}, 0), (\frac{1}{2}, 1)\}$.

$$d(A, B') = \max_{a_i \in A} \min_{b_j \in B'} d(a_i, b_j) = \frac{1}{2} \tag{96}$$

$$d(B', A) = \max_{b_j \in B'} \min_{a_i \in A} d(b_j, a_i) = \frac{1}{2} \tag{97}$$

$$h(A, B') = \max\{d(A, B'), d(B', A)\} = \frac{1}{2} \tag{98}$$

So

$$H^2(A,B) \tag{99}$$

$$=H^2(B,A) \tag{100}$$

$$=\inf_{t\in\mathbb{R}^2}\inf_{\theta} h(B^\theta+t,A) \tag{101}$$

$$=\inf_{t\in\mathbb{R}^2}\inf_{\theta} h(A,B^\theta+t) \tag{102}$$

$$=\frac{1}{2} \tag{103}$$

3.2 Compute D(A,B)

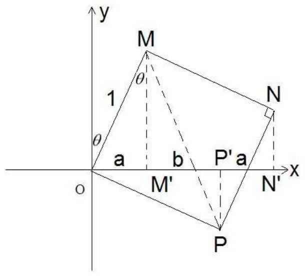

Figure 12: Diagram for computing the Yau-Hausdorff distance

First we compute $\sup_\theta \inf_\varphi H^1(P_x(A^\theta), P_x(B^\varphi))$. Without loss of generality we may assume $0 \le \theta \le \frac{\pi}{2}$.

Let the projection of M,N,P after rotation θ be M',N',P' (Fig.12).

Let $a = OM' = \sin\theta, b = M'P'$, then $P'N' = \sin\theta = a$.

Next we prove that $\inf_\varphi H^1(P_x(A^\theta), P_x(B^\varphi)) = \frac{1}{2}\min\{a,b\}$.

Assume $a \le b$, draw four disks of radius $\frac{1}{2}a$ centered at O, M', N', P', denoted as $C_O, C_{M'}, C_{N'}, C_{P'}$.

Because there are no more than three points in the projection of B^φ, there must be a disk that does not contain any point of $P_x(B^\varphi)$. So $H^1(P_x(A^\theta), P_x(B^\varphi)) \ge \frac{1}{2}a$, for any rotation φ.

We then take a rigid motion φ_0, s.t. $P_x(B^{\varphi_0}) = \{O, M', N'\}$.

Take $t = -\frac{1}{2}a$, and translate $P_x(B^{\varphi_0})$ by t.

Assume $P_x(B^{\varphi_0}) - \frac{1}{2}a = \{O'', M'', N''\}$. We can see that the Hausdorff distance

between $P_x(A^\theta)$ and $P_x(B^{\varphi_0}) - \frac{1}{2}a$ is $\frac{1}{2}a$. So

$$H^1(P_x(A^\theta), P_x(B^{\varphi_0})) = \frac{1}{2}a \tag{104}$$

$$\inf_{\varphi} H^1(P_x(A^\theta), P_x(B^{\varphi})) = \frac{1}{2}a = \frac{1}{2}\min\{a, b\} \tag{105}$$

Assume $b \leq a$, we can prove equation (105) in the same way.

Now we compute $\sup_\theta \inf_\varphi H^1(P_x(A^\theta), P_x(B^\varphi))$, it is equal to $\frac{1}{2}\sup_\theta \min\{a, b\}$. We can see that $\min\{a, b\}$ achieves the maximum for θ if and only if $a = b$, because if one of the values of $\{a, b\}$ increases, the other will decrease. Assume that the rotation of A is θ_0, s.t. a=b.

So

$$a = OM \sin\theta_0 = \sin\theta_0, \angle OMM' = \theta_0 \tag{106}$$

$$\angle M'MP = \angle OMP - \angle OMM' = \frac{\pi}{4} - \theta_0 \tag{107}$$

$$\begin{aligned} b &= MP \sin\angle M'MP && (108)\\ &= \sqrt{2}\sin(\frac{\pi}{4} - \theta_0) && (109)\\ &= \sqrt{2}(\frac{\sqrt{2}}{2}\cos\theta_0 - \frac{\sqrt{2}}{2}\sin\theta_0) && (110)\\ &= \cos\theta_0 - \sin\theta_0 && (111) \end{aligned}$$

$$\begin{aligned} & a = b && (112)\\ \Longrightarrow & \sin\theta_0 = \cos\theta_0 - \sin\theta_0 && (113)\\ \Longrightarrow & \cos\theta_0 = 2\sin\theta_0 && (114)\\ \Longrightarrow & \sin\theta_0 = \frac{\sqrt{5}}{5}, \cos\theta_0 = \frac{2\sqrt{5}}{5} && (115) \end{aligned}$$

So $a = b = \sin\theta_0 = \frac{\sqrt{5}}{5}$.

$$\begin{aligned}
&\sup_{\theta}\inf_{\varphi} H^1(P_x(A^\theta), P_x(B^\varphi)) && (116)\\
=&\inf_{\varphi} H^1(P_x(A^{\theta_0}), P_x(B^\varphi)) && (117)\\
=&\frac{1}{2}\min\{\ \frac{\sqrt{5}}{5}, \frac{\sqrt{5}}{5}\} && (118)\\
=&\frac{\sqrt{5}}{10} && (119)
\end{aligned}$$

Similarly we can prove that

$$\sup_{\varphi}\inf_{\theta} H^1(P_x(A^\theta), P_x(B^\varphi)) < \frac{\sqrt{5}}{10} \tag{120}$$

$$\begin{aligned}
&D(A, B) && (121)\\
=&\max\{\sup_{\theta}\inf_{\varphi} H^1(P_x(A^\theta), P_x(B^\varphi)), \sup_{\varphi}\inf_{\theta} H^1(P_x(A^\theta), P_x(B^\varphi))\} && (122)\\
=&\frac{\sqrt{5}}{10} && (123)
\end{aligned}$$
